# Recent educational tools in anaesthesiology residency training programs aligned with the European training requirements

**DOI:** 10.1097/EA9.0000000000000058

**Published:** 2024-08-08

**Authors:** Igor Abramovich, Iulia Crisan, Alessandro Scudellari, Federico Bilotta

**Affiliations:** From the Department for Anaesthesiology and Intensive Care Medicine (CCM/CVK), Charité - Universitätsmedizin Berlin, Berlin, Deutschland (IA), Department for Emergency Medicine, Universitätsspital Zürich, Zurich, Switzerland (IC), Department of Anaesthesia, Addenbrooke's Hospital, Cambridge, United Kingdom (AS) and Department of Anesthesiology, Critical Care and Pain Medicine, Policlinico Umberto I, University ‘La Sapienza’, Rome, Italy (FB)

Editor,

In recent years, various initiatives have aimed to strengthen the interconnection of training programs across Europe. Beyond standardising training durations, ensuring training quality has become a key focus. The European Board of Anaesthesiology of the European Union of Medical Specialists (EBA-UEMS) recently revised its European training requirements (ETR), adding a section on education programs and trainers, detailing methods for guiding and evaluating residents.^1^

The ETR emphasises outcomes, advancing residents based on acquired competencies rather than attempts or time spent. Competence is the overall ability to perform proficiently, while competency refers to specific skills needed for effective job execution.

Trainers accustomed to time-based education, now face the challenge of creating competency-based training environments. Success relies on skilled trainers within institutions prioritising training quality.

This shift is supported by both trainers and residents, who seek a robust evaluation framework.^2^ Below, we outline five areas with significant advancements in residency training.

Entrustable Professional Activities (EPAs) are specific tasks and responsibilities that residents should be able to perform effectively and independently once they reach a certain competence level on a predefined scale. EPAs represent a shift towards competency-based medical education, emphasising the application of knowledge and skills in real-world contexts. They are pivotal in anaesthesiology training, providing a framework to assess and entrust residents with essential tasks and determine the required supervision level.^3^

EPAs translate competencies into tangible clinical practice manifestations, typically encompassing a group of integrated competencies within a single EPA. For example, an EPA for airway management in a typical patient scenario includes not only practical skills like intubation but also medical knowledge, the ability to assess the patient's airway and experience in evaluating the effectiveness of airway management interventions.

In anaesthesiology, EPAs have been systematically developed and experimentally integrated into residency programs worldwide. Examples include thorough pre-operative patient assessment and procedural skills for managing an anticipated difficult airway.

Traditionally, residency programs primarily relied on summative examinations, often limited to one or two high-stake assessments throughout the training period. However, these examinations, focused on memorisation, provided little room for ongoing feedback and lacked the capacity to measure clinical competence effectively.

Responding to this global call for continuous feedback, Workplace-Based Assessments (WBA) have emerged as a vital solution.^4^ The ETR lists the following WBAs.

Direct Observation of Procedural Skills (DOPS) serves as an evaluative mechanism centred on the direct observation of a resident's performance in practical anaesthetic skills, such as ultrasound-guided venous access.

Anaesthetic Clinical Evaluation Exercise (A-CEX), functions as an evaluative tool intended to assess a resident's performance within the context of a clinical case.

Anaesthetic List Assessment Tool (ALMAT), formulated as an assessment instrument designed to appraise a resident's performance across a series of anaesthetic cases.

Case Based Discussion (CBD) entails a retrospective analysis and discussion of a specific anaesthetic case.

Each of those are designed to assess distinct facets of competence within the domain of anaesthesiology. This approach not only addresses the limitations of traditional examinations but also provides essential feedback for residents’ development, ensuring their preparedness for actual practice postgraduation.

Simulation-Based Medical Education (SBME) complement EPAs and WBAs by offering a controlled environment for learners to practice, refine and demonstrate competencies, facilitating targeted assessment and constructive feedback. This approach is particularly useful for preparing practitioners for challenging and unforeseen situations, such as airway emergencies.^5^

Despite varying implementation across European anaesthesiology residency programs, SBME's significance in residency training remains paramount. SBME enables systematic performance evaluation and constructive feedback, supporting the application of the latest guidelines and protocols. Recent developments have defined templates for learning objectives, instructional strategies and assessment methods to integrate SBME into anaesthesiology training. SBME assesses both procedural and nontechnical skills, including the application of advanced life support algorithms.

The completion of WBAs, EPAs and simulation courses alone does not ensure comprehensive residency training, as evaluating technical proficiency, nontechnical skills and professional attitudes requires more. An electronic portfolio integrates these elements, providing a digital framework to systematically capture, assess and reflect on residents’ competencies and achievements in real-world clinical settings, benefiting residents and trainers while facilitating mobility in Europe. Residents can efficiently track their training progress and engage in reflective learning, fostering self-awareness. Trainers gain a tool for assessments and dynamic curriculum management, ensuring targeted feedback and diverse clinical exposure. This comprehensive, data-driven approach supports professional development and facilitates smoother transitions for residents pursuing opportunities abroad or European exchanges. An electronic portfolio for anaesthesiology may include case summaries, procedural logs, reflections on clinical experiences, multimedia presentations, professional development plans, quality improvement projects, feedback and evaluations, certificates and awards, learning plans, assessments and mentorship activities.^6^

Research activities are essential in European anaesthesiology training programs, fostering critical thinking and evidence-based practice.^7^ Many programs require residents to contribute to the scientific literature, emphasising the importance of academic discourse and staying current with advancements in the field. Publishing scientific work is often necessary for program completion, enhancing educational experience and encouraging lifelong learning. Research endeavours can be optimised through specialised educational curricula and effective journal clubs during residency.

Implementing these tools presents challenges, as evidenced by some countries’ experiences, but it is crucial for standardising anaesthesiology residency training across Europe. This standardisation enhances uniformity, reliability and quality of training programs, facilitating the comparability of training regimens and promoting greater mobility within the European medical community. National societies should actively support these tools and advocate for their adoption. This collective effort would ensure integration and assesses the impact on training quality, establishing a framework for effective implementation.

Promoting these tools aligns with residents’ interests and streamlines comparing and evaluating training programs. Shifting to quantitative outcome measurement enhances assessment efficiency and training program effectiveness.

In conclusion, integrating these tools promises increased uniformity, reliability and mobility in anaesthesiology residency training. The collective support from individual programs, residents and national societies is crucial for their success, marking a significant advancement in anaesthesiology education.
